# Changes in peripapillary and subfoveal choroidal thickness in patients with central retinal vein occlusion

**DOI:** 10.1371/journal.pone.0255182

**Published:** 2021-08-20

**Authors:** Hae Min Kang, Jeong Hoon Choi, Hyoung Jun Koh, Sung Chul Lee

**Affiliations:** 1 Department of Ophthalmology, Catholic Kwandong University College of Medicine, Gangneung, Gangwon-do, South Korea; 2 Choikang Eye Clinic, Seoul, Republic of Korea; 3 Department of Ophthalmology, Yonsei University College of Medicine, Seoul, Republic of Korea; 4 Department of Ophthalmology, Konyang University College of Medicine, Daejeon, Republic of Korea; Sung Kyun Kwan University School of Medicine at Samsung Medical Center Cancer Center, REPUBLIC OF KOREA

## Abstract

**Purpose:**

We sought to evaluate changes of mean peripapillary choroidal thickness (PCT) and subfoveal choroidal thickness (SFCT) over 12 months in patients with unilateral central retinal vein occlusion (CRVO).

**Methods:**

Our retrospective, observational study included 19 patients with treatment-naïve, unilateral CRVO who completed at least 12 months of follow-up period. Mean PCT and mean SFCT in CRVO-affected eyes and unaffected contralateral eyes were measured at each follow-up visit, and then compared. Differences between baseline and 12 months (ΔSFCT and ΔPCT) and percentage changes (ΔSFCT or ΔPCT/baseline×100%) were determined. We also investigated the predictive factors for visual outcome in the CRVO-affected eyes.

**Results:**

In the CRVO-affected eyes, mean PCT was 146.7±41.9 μm at baseline, and 106.5±24.2 μm at 12 months (P < 0.001). Mean PCT of the contralateral eyes was 129.8±42.6 μm at baseline and 124.6±39.7 μm at 12 months (P = 0.089). Mean SFCT of CRVO-affected eyes was 225.8±77.9 μm at baseline, and 199.4±66.6 μm at 12 months (P = 0.009). Mean SFCT of the contralateral eyes was 218.4±83.0 μm at baseline, and 208.4±78.1 μm at 12 months (P = 0.089). Δ PCT was -41.6±25.3 μm in the CRVO-affected eyes, and -5.2±5.8 μm in the contralateral eyes (P<0.001). % PCT was -24.9±14.0% in the CRVO-affected eyes, and -4.0±0.4% in the contralateral eyes (P = 0.001). Δ SFCT was -26.4±24.6 μm in the CRVO-affected eyes, and -9.5±16.7μm in the contralateral eyes (P = 0.016). % SFCT was -10.4±9.8% in the CRVO-affected eyes, and -3.4±6.4% in the contralateral eyes (P = 0.015). Among the various factors, BCVA at baseline (β = 0.797, P = 0.001) and % SFCT (β = 0.712, P = 0.001) were significantly associated with visual outcome at 12 months in the CRVO-affected eyes.

**Conclusion:**

Both peripapillary and subfoveal choroidal thickness reduced significantly over 12 months in the CRVO-affected eyes, but not in the contralateral eyes. In addition, the absolute reduction amount and reduction ratio of PCT and SFCT were significantly greater in the CRVO-affected eyes than the contralateral eyes.

## Introduction

Central retinal vein occlusion (CRVO) is a vision-threatening retinal vascular disease that can lead to permanent vision loss. CRVO occurs predominantly in individuals over age 65 [1,2] and has risk factors that include systemic vascular diseases, such as diabetes and hypertension [2–4] while younger patients with CRVO may have underlying hypercoagulative disease or inflammatory disease [5–7]. Other risk factors for CRVO include glaucoma [8–10] and sleep apnea [11]^.^ The estimated prevalence of CRVO is approximately 0.1 to 0.4% and shows unilateral preference [1,12]. Several devastating complications can result from CRVO, including macular edema, retinal neovascularization, vitreous hemorrhage, and neovascular glaucoma, which can lead to permanent visual loss [1].

Despite its somewhat devastating prognosis, the precise pathogenesis of CRVO is still under investigation. Anatomic variations at the level of the lamina cribrosa may be important in CRVO pathogenesis, as a thrombus occluding the lumen of the central retinal vein at or just proximal to the lamina cribrosa, a meshwork supporting the optic nerve head (ONH), was identified in a histopathological study [13]. Because the central retinal vein and central retinal artery penetrate the lamina cribrosa [14], these vessels are naturally compressed as they cross through the rigid, sieve-like openings in the lamina cribrosa [1]. Mechanical stretching of the lamina cribrosa may further compress both vessels, leading to subsequent impingement on the central retinal vein [1]. The central retinal vein can also be compressed by an atherosclerotic central retinal artery or may be primarily occluded by inflammation [1]. Some studies have postulated that hemodynamic alterations induce stagnation of blood flow in the vein and result in primary thrombus formation in susceptible eyes [15,16].

Although CRVO primarily affects the inner retina, recent studies have investigated concomitant changes in macular choroidal thickness, suggesting that the macular choroid in CRVO may be altered in association with retinal ischemia [17–21]. Notably, macular choroidal thickness at the time of CRVO diagnosis may be useful for predicting clinical outcomes [22,23]. However, changes in the peripapillary choroid have not been explored in patients with CRVO, although the ONH (including the lamina cribrosa) is the main site of CRVO occurrence. Our study group previously investigated changes in the peripapillary choroid in various retinal vascular diseases, including unilateral branch retinal vein occlusion [24–26] and diabetic retinopathy [27]. The mean PCT was significantly decreased in the BRVO-affected eyes and unaffected contralateral eyes, although the amount of reduction was significantly greater in the BRVO-affected eyes [24]. Mean PCT was significantly thicker in advanced diabetic retinopathy, and then significantly decreased after panretinal photocoagulation [27].

Thus, in this study, we aimed to examine changes in mean peripapillary choroid thickness (PCT) of patients with treatment-naïve unilateral CRVO over a 12-month follow-up period, along with mean subfoveal choroidal thickness (SFCT) and mean retinal nerve fiber layer (RNFL) thickness. We also compared changes in mean PCT, mean SFCT, mean CMT, and mean RNFL thickness between the CRVO-affected eyes and the unaffected contralateral eyes, and investigated the possible factors associated with clinical outcomes in the CRVO-affected eyes.

## Methods

### Study population

This retrospective study was performed at International St. Mary’s Hospital, Catholic Kwandong University College of Medicine. The study protocol and waiver for informed consent were approved by the Institutional Review Board of International St. Mary’s Hospital, Catholic Kwandong University, and adhered to all tenets of the Declaration of Helsinki. The requirement for informed consent from each patient was waived due to the retrospective nature of the investigation. All the data were fully anonymized, and saved in the secured computer.

We reviewed medical records of treatment-naïve patients with unilateral CRVO who were seen at the hospital between January 2015 and December 2019. Inclusion criteria were as follows: 1) treatment-naïve patients diagnosed with CRVO by fluorescein angiography (FA) and 2) patients with regular follow-up visit times (between 9:00 and 11:00 A.M. or 2:00 and 4:00 P.M.) to minimize diurnal variation. Exclusion criteria were as follows: 1) patients with a history of intraocular pressure of ≥ 22 mmHg or presence of glaucoma at the time of CRVO diagnosis and 2) patients whose eyes exhibited severe media opacities, such as vitreous hemorrhage, that would obscure enhanced depth imaging-optical coherence tomography (EDI-OCT) of the retina and choroidal structures. All patients received 1.25 mg/0.05 ml bevacizumab (Avastin; Genentech/Roche, San Francisco, CA) by intravitreal injection for macular edema associated with CRVO.

We classified CRVO patients into one of two subgroups according to their perfusion status: perfused or nonperfused CRVO. Perfused CRVO is defined as fewer than 10 disc areas of retinal capillary nonperfusion upon FA, while nonperfused CRVO is defined as 10 or more such disc areas.^1^

The primary outcome measure of this study was to evaluate the changes of mean PCT during 12-month follow-up period in the CRVO-affected eyes, and compare to those of unaffected contralateral eyes. The secondary outcome measure was to find out the possibl impact of choroidal and macular changes on the visual outcome and the numbers of intravitreal injections in the patients with CRVO.

### Ophthalmologic examination of the study population

Ophthalmologic examination (e.g., slit lamp examination, intraocular pressure measurement using a non-contact tonometer, fundus examination) were performed for each patient. The refractive error value was measured for each eye using an autorefractor and converted to spherical equivalents [diopters (D)]. Best-corrected visual acuity (BCVA) was measured for each follow-up by using decimal visual acuity chart, and then converted to the logarithm of the minimum angle of resolution (logMAR). Multi-modal imaging studies included FA, fundus auto-fluorescence, and spectral domain OCT (SD OCT) (Spectralis; Heidelberg Engineering, Heidelberg, Germany) with EDI. FA was performed using the Heidelberg Retina Angiograph system (HRA-2; Heidelberg Engineering) with a confocal scanning laser ophthalmoscope. More details of SD OCT with EDI are described below.

### Retinochoroidal evaluation by using spectral domain optical coherence tomography

Measurement of chorioretinal structures was performed using SD OCT (Spectralis) with EDI as previously described [24–27]. Choroidal thickness was defined as the perpendicular distance from the outer border of the hyperreflective line, corresponding to the retinal pigment epithelium, to the chorioscleral interface.

For measuring PCT, a circular scan was centered on the ONH [3.40 mm diameter, “retinal nerve fiber layer (RNFL) circle scan”] in the peripapillary area. Using digital calipers provided in the Heidelberg Spectralis OCT software, PCT was measured at eight points (superior, superonasal, nasal, inferonasal, inferior, interotemporal, temporal, and superotemporal), and the average PCT was calculated for each patient.

For macular evaluation, serial cross-sectional horizontal scans approximately 121 μm apart in a 30 × 30° macular area were obtained. Single horizontal and vertical scans across the fovea were also obtained separately. To measure subfoveal choroidal thickness (SFCT), at least two high-quality horizontal and vertical scans across the fovea for each eye were obtained. Using digital calipers as described above, SFCT was measured horizontally and vertically at the subfoveal region in each trans-sectional image and then averaged. Two independent observers (HMK and JHC) blinded to the clinical data of each patient performed the PCT and SFCT measurements of each eye.

RNFL thickness and central macular thickness (CMT) were automatically measured by OCT. Using “RNFL circle scan” as described above, RNFL thickness in six sectors (temporal, superotemporal, superonasal, nasal, inferonasal, and inferotemporal) was measured and then automatically averaged by the Spectralis OCT software. CMT was defined as the mean retinal thickness in the central subfield, a region with a diameter of 1.0 mm around the fovea. The inner and the outer rings had diameters of 3.0 mm and 6.0 mm, respectively. All SD OCT images were reviewed for any possibility of segmentation errors.

After measurement of mean PCT, mean SFCT, mean CMT, and mean RNFL thickness, we calculated the difference of each eye (delta PCT, Δ PCT; delta SFCT, Δ SFCT; delta CMT, Δ CMT; and delta RNFL thickness, Δ RNFL thickness) as following: [the value at 12-month follow-up point–the value at baseline]. The ratio of the change of each value (percent PCT, %PCT; percent SFCT, % SFCT; percent CMT, % CMT; and percent RNFL thickness, % RNFL thickness) in each eye was also calculated as following: the difference between baseline and 12 months (Δ PCT, Δ SFCT, Δ CMT, or Δ RNFL thickness)/baseline value] x 100 (percentage; %).

### Statistical analysis

SPSS version 22.0 for Windows (IBM Corporation, Somers, NY, USA) was used for statistical analyses. Baseline characteristics, including age at the time of diagnosis and sex, were evaluated. The presence of hypertension, diabetes mellitus, cardiovascular disease (such as myocardial infarct), and cerebrovascular disease (such as ischemic stroke) was investigated. Comparisons of CRVO-affected eyes and unaffected contralateral eyes were performed using the Wilcoxon signed rank test for continuous variables. Repeated-measures analysis of variance was used to assess significant differences between repeatedly measured variables, including the mean best corrected visual acuity (BCVA), CMT, SFCT, PCT, and RNFL thickness. Pearson’s correlation analysis was used to investigate significant correlations between two factors. Multiple regression analysis was used to evaluate the possible correlation of baseline values with visual outcome and the total numbers of intravitreal injections in the CRVO-affected eyes. Mauchly’s test of sphericity and Kolmogorov-Smirnov analyses were used to confirm statistical validity. Results with P < 0.05 were considered statistically significant.

## Results

### Baseline characteristics of the study population

Nineteen patients with treatment-naïve unilateral CRVO were included in this retrospective, observational study. The mean age at the time of diagnosis was 64.4±17.0 (26 to 96) years, and 10 patients (52.6%) were male. The mean follow-up duration was 29.9±19.5 (12 to 66) months. Seven (38.4%) patients were diagnosed with ischemic CRVO. The study population received a mean of 2.3±0.9 (1 to 4) intravitreal bevacizumab injections during the follow-up period. Three patients received additional intravitreal triamcinolone acetonide injections, each 2 times during follow-up period. The total number of intravitreal injections was 2.7±1.3 times (1 to 6 times) in the CRVO-affected eyesDetailed baseline characteristics of the study population are shown in Table 1.

**Table 1:** Baseline characteristics of enrolled patients with unilateral CRVO.

**Sex (Male)**	10 (52.6%)
**Mean age at time of diagnosis**	64.4±17.0 (26 to 96)
**Symptom duration (weeks)**	11.2±2.6 (10 to 14)
**Perfused/Nonperfused CRVO**	7 (38.4%)/12 (61.6%)
**Diabetes mellitus**	6 (31.6%)
**Hypertension**	8 (42.1%)
**Myocardial infarction**	1 (5.3%)

### Changes of mean best-corrected visual acuity in the central retinal vein occlusion-affected eyes and unaffected contralateral eyes over 12 months

**Fig 1:**
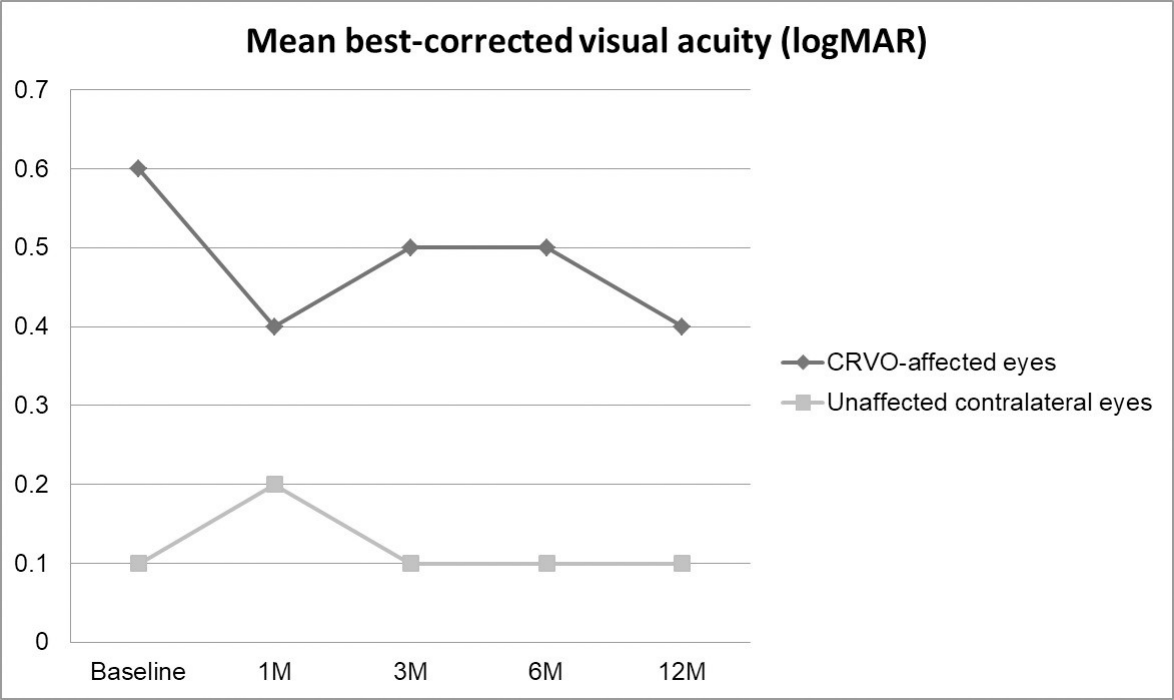
Changes of mean best-corrected visual acuity (BCVA) in central retinal vein occlusion (CRVO)-affected eyes and unaffected contralateral eyes over 12 months. The mean BCVA at each follow-up point was significantly worse in the CRVO-affected eyes than those in the unaffected contralateral eyes ((P<0.001 at baseline; P = 0.013 at 1 month; P = 0.008 at 3 months; P = 0.002 at 6 months; and P = 0.001 at 12 months).

The mean BCVA of CRVO-affected eyes was 0.6±0.3 logMAR (0.1 to 1.3 logMAR) at baseline and 0.4±0.4 logMAR (0 to 1 logMAR) at 12 months (P = 0.222). The mean BCVA of unaffected contralateral eyes was 0.1±0.1 logMAR (0 to 0.5 logMAR) at baseline and 0.1±0.1 logMAR (0 to 0.5 logMAR) at 12 months (P = 0.0.419). At each follow-up time point, the mean BCVA was significantly worse in CRVO-affected eyes than contralateral eyes (P<0.001 at baseline; P = 0.013 at 1 month; P = 0.008 at 3 months; P = 0.002 at 6 months; and P = 0.001 at 12 months). Changes in mean BCVA are depicted in Fig 1.

When compared with baseline, 12 eyes (63.2%) with CRVO showed 0.2 logMAR or more improve of BCVA when compared with baseline, 4 eyes (21.1%) with CRVO did 0.2 logMAR or more deterioration, and 3 eyes (15.7%) did less than 0.2 logMAR changes of BCVA at 12 months,

### Changes of mean central macular thickness in the central retinal vein occlusion-affected eyes and unaffected contralateral eyes over 12 months

**Fig 2:**
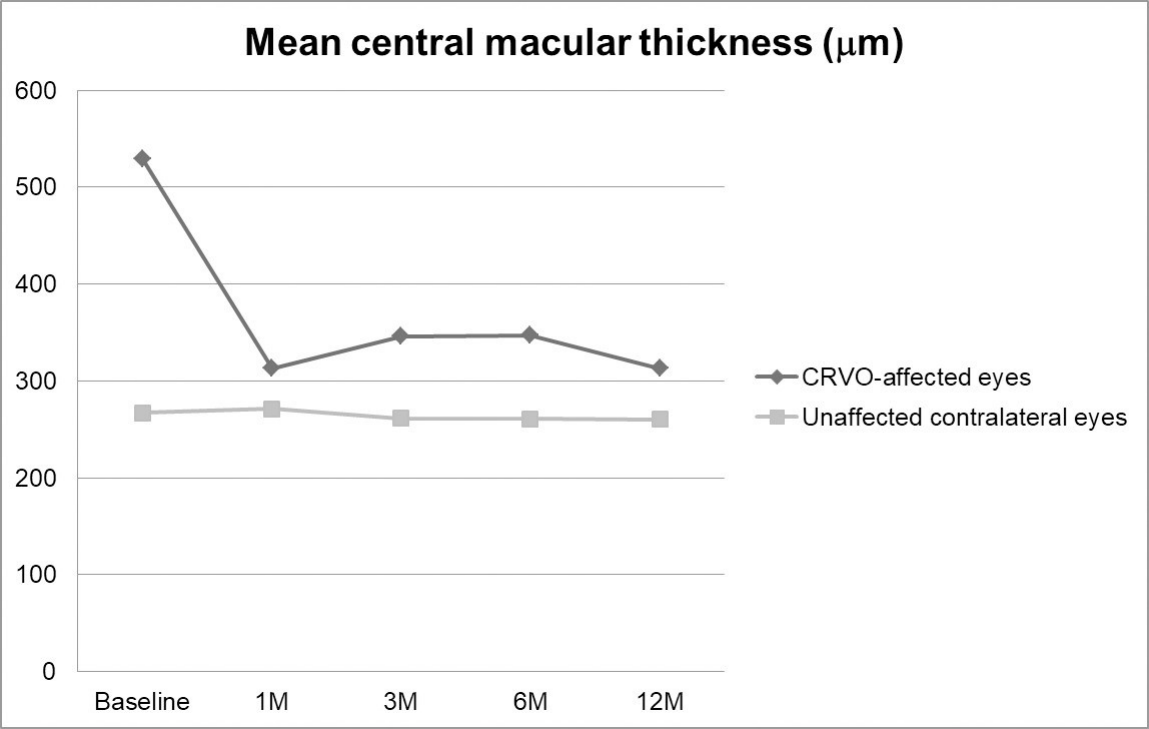
Changes in mean central macular thickness (CMT) in central retinal vein occlusion (CRVO)-affected eyes and unaffected contralateral eyes. The mean CMT at each follow-up point was significantly thicker in the CRVO-affected eyes than those in the unaffected contralateral eyes (P<0.001 at baseline; P = 0.017 at 1 month; P = 0.007 at 3 months; P = 0.005 at 6 months; and P = 0.027 at 12 months).

The mean CMT of CRVO-affected eyes was 529.4±132.2 μm (244.0 to 765.0 μm) at baseline and 312.7±108.9 μm (202.0 to 663.0 μm) at 12 months (P<0.001). For unaffected contralateral eyes, the mean CMT was 266.7±34.6 μm (188.0 to 435.0 μm) at baseline and 259.7±28.6 μm (184.0 to 261.4 μm) at 12 months (P<0.001). We found that the mean CMT was significantly thicker in CRVO-affected eyes than in contralateral eyes at each follow-up time point (P<0.001 at baseline; P = 0.017 at 1 month; P = 0.007 at 3 months; P = 0.005 at 6 months; and P = 0.027 at 12 months). Changes in mean CMT are depicted in Fig 2.

### Changes of mean subfoveal choroidal thickness in the central retinal vein occlusion-affected eyes and unaffected contralateral eyes over 12 months

**Fig 3:**
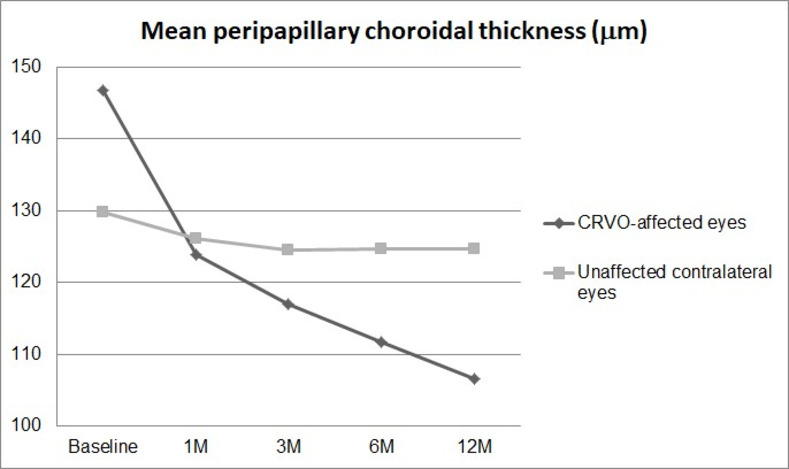
Changes in mean subfoveal choroidal thickness (SFCT) in central retinal vein occlusion (CRVO)-affected eyes and unaffected contralateral eyes. The mean SFCT was not significantly different between the CRVO-affected eyes and the contralateral eyes at each follow-up point (P = 0.782 at baseline; P = 0.815 at 1 month; P = 0.872 at 3 months; P = 0.715 at 6 months; and P = 0.609 at 12 months).

The mean SFCT of CRVO-affected eyes was 225.8±77.9 μm (59.0 to 375.0 μm) at baseline and 199.4±66.6 μm (64.0 to 311.5 μm) at 12 months (P = 0.009). The mean SFCT of unaffected contralateral eyes was 218.4±83.0 μm (60.0 to 347.0 μm) at baseline and 208.4±78.1 μm (62.0 to 312.0 μm) at 12 months (P = 0.089). Mean SFCT values were not significantly different between CRVO-affected eyes and contralateral eyes at any follow-up time point (P = 0.782 at baseline; P = 0.815 at 1 month; P = 0.872 at 3 months; P = 0.715 at 6 months; and P = 0.609 at 12 months). Changes in mean SFCT are shown in Fig 3.

### Changes of mean peripapillary choroidal thickness in the central retinal vein occlusion-affected eyes and unaffected contralateral eyes over 12 months

**Fig 4:**
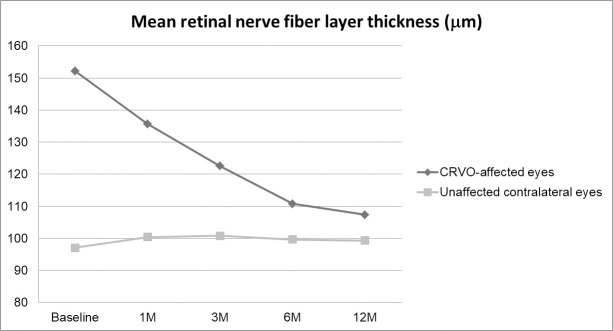
Changes in mean peripapillary choroidal thickness (PCT) in central retinal vein occlusion (CRVO)-affected eyes and unaffected contralateral eyes. The mean PCT was not significantly different between the CRVO-affected eyes and the contralateral eyes at each follow-up point (P = 0.314 at baseline; P = 0.782 at 1 month; P = 0.815 at 3 months; P = 0.693 at 6 months; and P = 0.343 at 12 months).

In the CRVO-affected eyes, the mean PCT was 146.7±41.9 μm (77.1 to 222.9 μm) at baseline, and 106.5±24.2 μm (66.6 to 154.8 μm) at 12 months (P < 0.001). The mean PCT of unaffected contralateral eyes was 129.8±42.6 μm (71.1 to 205.9 μm) at baseline and 124.6±39.7 μm (70.8 to 198.5 μm) at 12 months (P = 0.089). We did not observe any significant differences between mean PCT between CRVO-affected eyes and contralateral eyes at any follow-up time point (P = 0.314 at baseline; P = 0.782 at 1 month; P = 0.815 at 3 months; P = 0.693 at 6 months; and P = 0.343 at 12 months). Changes in mean PCT are presented in Fig 4.

### Changes of mean retinal nerve fiber layer thickness in the central retinal vein occlusion-affected eyes and unaffected contralateral eyes over 12 months

**Fig 5:**
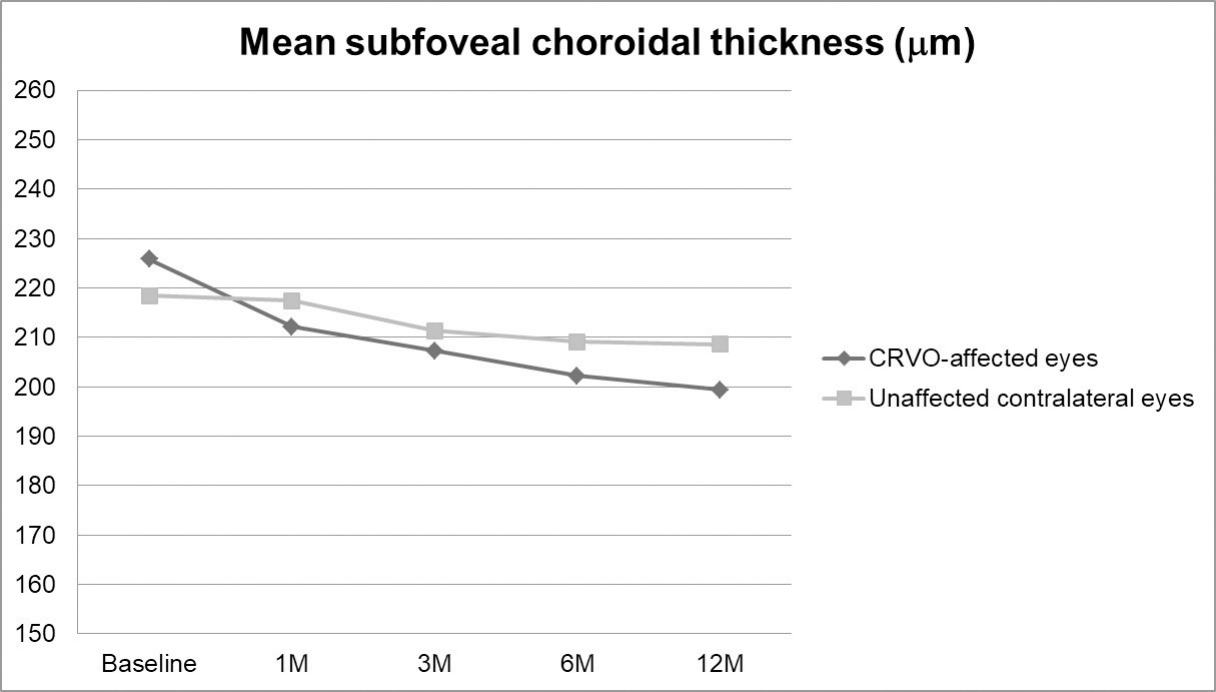
Changes in mean retinal nerve fiber layer (RNFL) thickness in central retinal vein occlusion (CRVO)-affected eyes and unaffected contralateral eyes. The mean RNFL thickness was significantly thicker in the CRVO-affected eyes than the contralateral eyes at baseline (P<0.001), at 1 month (P = 0.001), and at 3 months (P = 0.013). There was no significant difference at 6 months (P = 0.068) and at 12 months (P = 0.140).

The mean RNFL thickness of CRVO-affected eyes was 152.2±49.1 μm (85.0 to 243.0 μm) at baseline and 107.3±25.5 μm (64.0 to 175.0 μm) at 12 months (P = 0.006). The mean RNFL thickness of unaffected contralateral eyes was 97.1±14.7 μm (71.1 to 159.0 μm) at baseline and 99.3±13.3 μm (76.0 to 126.0 μm) at 12 months (P = 0.406). Notably, the mean RNFL thickness was significantly thicker in CRVO-affected eyes than in contralateral eyes at baseline (P<0.001), 1 month (P = 0.001), and 3 months (P = 0.013). However, we saw no significant difference at 6 months (P = 0.068) and at 12 months (P = 0.140), although the mean RNFL thickness at these time points was still greater in CRVO-affected eyes than in contralateral eyes. Fig 5 shows changes observed in mean RNFL thickness.

### Comparison of changes in the central retinal vein occlusion-affected eyes and the unaffected contralateral eyes

We calculated the mean changes of CMT, SFCT, PCT, and RNFL thickness in each CRVO-affected eye and unaffected contralateral eye between baseline and 12-month follow-up point. When compared, mean Δ PCT, Δ SFCT, Δ CMT, and Δ RNFL thickness were significantly larger in the CRVO-affected eyes than the unaffected contralateral eyes (P < 0.001; P = 0.016; P < 0.001; and P < 0.001, respectively). The ratio of changes, in that, % PCT, % SFCT, % CMT, and % RNFL thickness were also significantly larger in the CRVO-affected eyes than the unaffected contralateral eyes (P = 0.001; P = 0.015; P < 0.001; and P = 0.001, respectively). The detailed comparison is shown in Table 2.

**Table 2:** Comparison of changes between central retinal vein occlusion affected eyes and unaffected contralateral eyes.

	CRVO-affected eyes (N = 19)	Unaffected contralateral eyes (N = 19)	P value*
**Δ PCT (μm)**	-41.6±25.3	-5.2±5.8	< 0.001
**% PCT (%)**	-24.9±14.0	-4.0±0.4	< 0.001
**Δ SFCT (μm)**	-26.4±24.6	-9.5±16.7	0.016
**% SFCT (%)**	-10.4±9.8	-3.4±6.4	0.015
**Δ CMT (μm)**	-216.7±145.0	-38.2±21.2	< 0.001
**% CMT (%)**	-38.2±21.2	-0.7±2.9	< 0.001
**Δ RNFL thickness (μm)**	-44.9±39.1	-0.4±2.9	<0.001
**% RNFL thickness (%)**	-20.6±23.7	-0.8±2.2	0.001

Abbreviations: CMT, central macular thickness; PCT, peripapillary choroidal thickness; RNFL, retinal nerve fiber layer thickness; SFCT, subfoveal choroidal thickness.

*The Wilcoxon signed rank test was used for statistical analysis.

†Differences between baseline and 12 months (Δ) and percentage changes (Δ value/baseline×100%) were determined.

We also investigated any possible correlations: there were no significant correlations between Δ CMT and Δ SFCT (P = 0.746), between Δ CMT and Δ PCT (P = 0.556), between Δ CMT and Δ RNFL (P = 0.162), between Δ SFCT and Δ RNFL (P = 0.372), between Δ PCT and Δ RNFL (P = 0.203), and between Δ PCT and Δ SFCT (P = 0.623). There were significant correlations between % CMT and % RNFL (P = 0.038) and between % RNFL and % SFCT (P = 0.008), but not in between % CMT and % SFCT (P = 0.389), between % CMT and % PCT (P = 0.089), between % PCT and % RNFL (P = 0.275), and between % PCT and % SFCT (P = 0.093).

### Possible factors associated with visual outcome and the number of treatment in central retinal vein occlusion

We performed multiple regression analysis to investigate the possible factors associated with visual outcome at 12 months in the CRVO-affected eyes. Among the various factors, BCVA at baseline (β = 0.797, P = 0.001) and % SFCT (β = 0.712, P = 0.001) were significantly associated with visual outcome at 12 months in the CRVO-affected eyes. Detailed results of multiple regression analysis are shown in Table 3.

**Table 3:** Possible predictive factors for visual outcome at 12 months in the eyes with central retinal vein occlusion.

Factors	P value
**Sex**	0.628
**Age**	0.937
**CMT at baseline**	0.679
**Δ CMT**	0.365
**% CMT**	0.231
**Mean SFCT at baseline**	0.210
**Δ SFCT**	0.234
**Mean PCT at baseline**	0.836
**Δ PCT**	0.434
**% PCT**	0.373
**RNFL at baseline**	0.286
**Δ RNFL**	0.461
**% RNFL**	0.669

Abbreviations: CMT, central macular thickness; PCT, peripapillary choroidal thickness; RNFL, retinal nerve fiber layer thickness; SFCT, subfoveal choroidal thickness.

Differences between baseline and 12 months (Δ) and percentage changes (Δ value/baseline×100%) were determined.

## Discussion

In our study, we investigated changes in mean PCT over 12 months in patients with unilateral, treatment-naive CRVO. Mean PCT decreased significantly in CRVO-affected eyes over 12 months, however, mean PCT was not significantly change in unaffected contralateral eyes. This tendency was also applied to other values: mean SFCT, mean CMT, and mean RNFL thickness significantly decreased in the CRVO-affected eyes, whereas those of unaffected contralateral eyes did not over 12 months. After a gradual decrease, the mean SFCT and PCT of CRVO-affected eyes were ultimately less than those of contralateral eyes at 12 months, although the difference did not reach statistical significance.

Although mean PCT was not significantly different between CRVO-affected eyes and unaffected contralateral eyes, there were significant differences in mean Δ PCT and mean % PCT. In that, the changes of PCT during 12 months and the ratio of changes to the baseline values were significantly larger in the CRVO-affected eyes than the unaffected contralateral eyes. The same tendencies were shown in the changes of SFCT, CMT, and RNFL thickness: significantly larger changes were observed in the CRVO-affected eyes than the unaffected contralateral eyes between baseline and 12-month follow-up points. In addition, baseline BCVA and % SFCT were significantly associated with visual outcome at 12 months in the CRVO-affected eyes.

Although CRVO mainly affects the inner retina, recent studies have shown that retinal hypoxia due to CRVO further induces choroidal changes [17–23], as well as hyperpermeability and increased vascular endothelial growth factor (VEGF) expression in the inner retina [28]. VEGF produced in response to retinal ischemia seems to translocate to the choroid, and increased leakage from choroidal blood vessels leads to choroidal thickening, especially in the stromal area [22,29–31]. Retinal hypoxia also induces upregulation of angiogenic and vascular permeability factors, as well as increased production of inflammatory factors, such as inflammatory cytokines and macrophage attractants [32]. Increased intraocular VEGF concentrations stimulate production of nitric oxide, vessel dilation, and ocular blood flow [33–35]. One study also suggests that an increased volume of choroidal stroma in CRVO may result from a rush of extracellular fluid that failed to be removed due to impaired retinal venous outflow toward the choroid [19]. Because the choroid is a major component of the unconventional fluid outflow system, fluid ingress may induce choroidal swelling [36]. Consistent with previous studies [17–23], we found that both the mean PCT and SFCT were greater in CRVO-affected eyes than in unaffected contralateral eyes, although the difference did not reach statistical significance. Regardless, our findings suggest that choroidal thickening associated with CRVO not only affects the macula but also the peripapillary area.

Patients in our study received intravitreal bevacizumab injections to relieve CRVO-associated macular edema. After the injections, the mean CMT reduced significantly rover 12 months, although the mean BCVA did not significantly improve. Anti-VEGF agents have been shown to reduce choriocapillaris endothelial cell fenestrations, reducing leakage through such fenestrated vascular walls [37]. Thus, anti-VEGF agents ultimately reduce swelling of the choroidal stroma and normalize choroidal thickness. One study compared choroidal changes after administration of intravitreal anti-VEGF agents, dexamethasone implants, and triamcinolone acetonide and showed that these agents similarly reduce choroidal thickness in eyes with CRVO [38]. In our study, both mean PCT and SFCT were significantly reduced in patients after their first intravitreal bevacizumab injection, which is consistent with previous reports [17–23]. In addition, the changed amounts of PCT and SFCT were significantly greater in the CRVO-affected eyes when compared those values in the unaffected contralateral eyes. These results also support previous studies that anti-VEGF agents can effectively reduce choroidal thickness as well as macular thickness in CRVO patients [17–23]. In addition, our findings suggest that anti-VEGF agents can affect the peripapillary choroidal area as well as subfoveal area.

We did not observe significant differences between mean SFCT and PCT values in CRVO-affected eyes vs. contralateral eyes. In contrast, previous studies have shown that the mean SFCT is significantly greater in CRVO-affected eyes than in contralateral eyes [17–23]. In our study, mean SFCT and mean PCT at baseline were greater in CRVO-affected eyes than those in contralateral eyes, however, they did not reach clinical significances. These values gradually decreased in CRVO-affected eyes and were less than those in contralateral eyes during the follow-up period, although the difference was not statistically significant. The lack of significance may be due to the characteristics of our study population, which included both perfused and nearly 40% of nonperfused CRVO patients. Perfused CRVO is characterized by increased venous intraluminal pressure that favors outward leakage of fluid from affected vessels so that effects of both hypoxia and hydrostatic stress are likely involved in the development of macular edema [32]. Increased intraluminal pressure may also lead to secondary choroidal swelling in perfused CRVO, as well as VEGF-induced choroidal thickening. A thicker choroid in CRVO may be associated with higher VEGF levels. Because perfusion status and degree of macular edema may differ in each patient of our study population, the inclusion of CRVO patients with lower VEGF levels may also have influenced in our findings regarding the non-significant differences in SFCT and PCT in CRVO-affected vs. contralateral eyes. Thus, further studies that include a larger number of perfused and nonperfused CRVO patients is warranted to compare changes of mean SFCT and PCT between the two subgroups and deepen our understanding CRVO and its associated choroidal changes.

In our study population, mean RNFL thickness in CRVO-affected eyes was significantly greater than that in contralateral eyes at baseline, 1 month, and 3 months but not significantly different at 6 or 12 months. However, the mean PCT and RNFL thickness were significantly correlated at 6 months and 12 months in CRVO-affected eyes. We assume that profuse RNFL swelling during acute-phase CRVO gradually resolves and then mean PCT and RNFL thickness seem to be associated during the chronic phase. Although we did not investigate the clinical impact of changes in PCT and RNFL thickness on ONH function, other long-term prospective studies that incorporate a visual field test could demonstrate the impact of these changes on CRVO eye function.

In our study, we also investigated the possible factors associated with visual outcome in the CRVO-affected eyes. Multiple regression analysis showed that BCVA at baseline and % SFCT are significantly correlated with visual outcome at 12 months: better BCVA at baseline and more SFCT reduction from the baseline value are associated with better visual outcome in the CRVO-affected eyes. Previous studies have suggested that thicker SFCT at baseline may be a positive predictive factor for functional recovery after intravitreal anti-VEGF injections in the CRVO-affected eyes [22,23]. As we mentioned earlier, choroidal congestion in CRVO may be associated with increased VEGF levels due to retinal ischemia [33–36], and secondary effect from increased luminal pressure in perfused CRVO [32]. Thus, better perfusion status of CRVO and effective reduction of VEGF after intravitreal anti-VEGF injections may lead to better clinical outcome in the patients with CRVO.

Our study has several limitations, including a relatively small study population and the retrospective study design. Due to the small sample size, we could not perform subgroup analysis according to perfusion status. In addition, our results suggest that mean SFCT and PCT in CRVO-affected eyes were lower than those in contralateral eyes at 12 months, but a future study with a longer follow-up period is needed to investigate if choroidal thinning continues to progress in CRVO-affected eyes and to determine its possible impact on clinical outcomes, both functional and anatomical. Further studies with both a larger study population and a functional evaluation component will enhance our knowledge of CRVO according to disease subset.

In conclusion, both peripapillary and subfoveal choroidal thickness reduced significantly over 12 months in CRVO-affected eyes, but not in contralateral eyes. Althugh mean PCT and mean SFCT were not significantly different between CRVO-affected eyes and those unaffected contralateral eyes, the absolute reduction amount and reduction ratio of both PCT and SFCT were significantly greater in CRVO-affected eyes than unaffected contralateral eyes. Because baseline BCVA and ratio of SFCT reduction were significantly associated with visual outcome in the CRVO-affected eyes, further investigations on the clinical impact of peripapillary and subfoveal choroid in CRVO may be helpful for managing these patients.
